# Current best practices and future opportunities for reproducible findings using large-scale neuroimaging in psychiatry

**DOI:** 10.1038/s41386-024-01938-8

**Published:** 2024-08-08

**Authors:** Neda Jahanshad, Petra Lenzini, Janine Bijsterbosch

**Affiliations:** 1 Mark and Mary Stevens Neuroimaging & Informatics Institute, Keck School of Medicine of USC, Marina del Rey, CA, 90292 USA; 2 Department of Radiology, Washington University School of Medicine, Saint Louis, MO 63110 USA

**Keywords:** Neuroscience, Psychology

## Abstract

Research into the brain basis of psychopathology is challenging due to the heterogeneity of psychiatric disorders, extensive comorbidities, underdiagnosis or overdiagnosis, multifaceted interactions with genetics and life experiences, and the highly multivariate nature of neural correlates. Therefore, increasingly larger datasets that measure more variables in larger cohorts are needed to gain insights. In this review, we present current “best practice” approaches for using existing databases, collecting and sharing new repositories for big data analyses, and future directions for big data in neuroimaging and psychiatry with an emphasis on contributing to collaborative efforts and the challenges of multi-study data analysis.

## Introduction

Research into the brain basis of psychopathology is challenging due to the heterogeneity of psychiatric disorders, extensive comorbidities, underdiagnosis or overdiagnosis, multifaceted interactions with genetics and life experiences, and the highly multivariate nature of neural correlates. Therefore, increasingly larger datasets that measure more variables in larger populations are needed to gain insights. Neuroimaging datasets have steadily increased in sample size from 12 participants in the 1990 s and early 2000 s [1] to 100+ participants in recent years [2], with mega studies going up to 1000 (Human Connectome Project; HCP [3]), 10,000 (Adolescent Brain Cognitive Development; ABCD [4]), and even 100,000 neuroimaging participants (UK Biobank [5]). These larger neuroimaging datasets offer opportunities to gain novel insights into psychopathology, but also bring along new challenges. The aim of this paper is to provide a practical guide for users and creators of existing and future big datasets. In the rest of the introduction we briefly summarize the what, why, and how of big data. Afterwards, we discuss five best practice categories for researchers using existing big data and five best practice categories for researchers planning to acquire or contribute to big data.

### What are big data in psychiatry?

Although big data have been defined in a number of different ways (including V-model descriptions covering volume and variety [6]), for the purposes of this article we will consider big data to refer to datasets that are larger and more complex than traditional studies. As such, what constitutes big data is a moving target and the respective sample sizes described in Table 1 may change over time. Although data size is most commonly measured in terms of the number of participants (N), datasets can be “big” in other dimensions including longitudinal precision functional mapping data that obtains many repeated measures often across a wide range of constructs in a small number of individuals (e.g., the Midnight Scan Club) [7, 8]. The large-N definition of big data includes prospectively planned samples, retrospectively combined samples, and epidemiological samples (Table 1). Notably, the majority of currently available big neuroimaging data in psychiatry are observational in nature and do not include experimentally controlled interventions. Nevertheless, randomized controlled trials (RCTs) are increasingly mandated to share data as a result of journal and/or funding policies [9, 10], creating new opportunities for data pooling and reanalysis [11, 12]. Even traditionally sized datasets often can and should be shared [13, 14], so that they can contribute to retrospective big datasets. As such, many of the recommendations covered in this article are relevant across all study types.

**Table 1:** Overview of study categories as a function of sample size.

	Precision data	Traditional data	Prospective big data	Retrospective big data	Epidemiological data
Approximate sample size	1–50	50–250	500–5000	1000+	10,000+
Acquisition	Single laboratory	Single laboratory	Consortium	Consortium	Consortium
Cohort	Precision mapping in individuals	Strict inclusion & exclusion criteria	Some criteria for healthy or disease-specific cohorts	Smaller studies (*N* < 1000) combined retrospectively	Population representative (very few exclusion criteria)
Study protocol	Many (10+) repeated measurements	Dedicated for specific research question/ intervention	Multimodal covering wide range of popular data types	Post-hoc harmonization or meta analysis of existing data	Multi-omic covering as wide a range as possible
Benefits	Single subject analysis	Full control over study design	Well powered for small effect sizes	Expandable by adding additional datasets	Transdiagnostic research

Notably, sample size ranges represent approximate ranges based on the current literature. Some sample size ranges are not covered for practical reasons. Specifically, *N* = 250–1000 is typically too large to be feasible for a single laboratory funded by traditional grant mechanisms, yet too small to be boosted by the benefits of big data. Furthermore, *N* = 5000–10,000 only provides an incremental power boost beyond the big data sample size, yet also cannot achieve the population representation required for epidemiological data.

### Why are big data useful?

The most important reason that bigger datasets are needed is related to statistical power. The statistical power to detect an effect of interest is driven by three factors, namely: (i) the magnitude of the “true” effect in the population, (ii) the size of the sample used to study the effect, and (iii) the statistical significance criterion, which includes the multiple comparison burden. Underpowered studies suffer from sampling variability such that the value of an observed statistical result can vary drastically between study samples [15]. Such sampling variability in underpowered studies has contributed to what has been called the replication crisis [16, 17]. Sampling variability combined with publication bias [18] has created the illusion of strong effect sizes, but recent studies are increasingly demonstrating that the magnitude of “true” brain-behavior effects in the population may be much smaller than previously thought [19]. Small magnitudes of “true” effect in the population may arise from unreliable diagnostic labels [20–22], self report bias [23, 24], low reliability of common emotional and attention tasks [25, 26], and low reliability of neuroimaging measures [27]. Specifically, the combined reliability of the neuroimaging and clinical measures place an upper limit on the strength of brain-symptom associations that can be observed, because a variable cannot have a higher correlation with another variable than it does with itself [28, 29]. Low effect sizes in well powered studies therefore do not necessarily imply that the investigated relationships are clinically meaningless or trivial, and indeed it is possible that the underlying constructs – free from measurement error and bias – may be more tightly associated. As such, focusing on rigorously testing the replicability of findings and improving the reliability of both neuroimaging and clinical measures should be a priority.

In addition to the statistical power offered by larger sample sizes, big data offer important additional advantages for psychiatric research. These advantages can broadly be summarized into four categories, namely: generalizability, multi-omics, transdiagnostic research, and unique cohorts. Box 1 summarizes broad categories of research opportunities that are aided or newly enabled by big data, including some select example studies. Indeed, the value of big data for psychiatric research is increasingly recognized as evidenced by a dedicated working group in the National Institute of Health (National Advisory Mental Health Council Workgroup on High-Dimensional Datasets).

Box 1 Example research opportunities enabled by big data Replicability/Reproducibility/Generalizability

- Replication of findings in held-out data and/or across multiple independent datasets [133]
- Generalizability of findings in underrepresented communities not traditionally included in study samples [134]
- Inclusion of individuals with symptom presentation below the clinical threshold [135]
- Inclusion of individuals with undiagnosed/under-diagnosed symptoms

Multi-omics

- Leveraging and integrating information across multiple data modalities [136], such as:
  - Genomics
  - Neuroimaging
  - Clinical/symptom information
  - Cognition
  - Wearables (e.g., activity trackers)
  - Lifestyle information (e.g., diet, exercise, social support)
  - Socio-economic information (e.g., income, area deprivation index)
  - Biological markers (blood, saliva, urine)

Transdiagnostic research

- Transdiagnostic investigation of symptom constructs that are shared across many diagnoses [137]
- Investigations of comorbid disease presentations
- Determination of the disease specificity of biomarkers [138]
- Investigations into disease heterogeneity, subtypes/ biotypes, and latent profiles [139]

Unique cohorts

- Investigations into tightly stratified groups (e.g., defined based on symptoms or genomics) [140]
- Dissociation of factors that are typically collinear (e.g., state & trait symptomatology) [141]

### How do big data work?

Where, how, and with whom big data are shared varies substantially. Some data sharing frameworks adopt a consortium or working group approach that requires collaboration to get data access (e.g., Enhancing Neuro Imaging Genetics through Meta Analysis; ENIGMA [30]), others share freely (e.g., Human Connectome Project), and yet others offer a tiered fee structure to support the sustainability of the resource (e.g., UK Biobank). Importantly, data sharing is increasingly mandated by funding agencies [31–33]. Although the detailed access procedures vary substantially from one dataset to another, several of the recommendations below provide a general introduction to concepts and procedures for accessing existing data and sharing your data.

In summary, big data are an important new tool in the overall toolkit used to study the brain basis of psychopathology. The goal of this paper is to discuss ideas, suggestions, and opportunities for making the most of this new big data tool. This work is not intended to be prescriptive, but rather to encourage awareness and inspire discussion and innovation in the field.

## Using existing big data

Researchers across most fields increasingly have the option to access and work with existing data resources. This section lays out five practical recommendations for researchers to make the most of these existing big data resources. For study-specific guides, please note that additional resources are available (e.g., ABCD [34]).

### Recommendation 1: Compliance with data usage requirements

#### Understand and comply with contractual agreements for secondary data research

Users who wish to access existing datasets are often required to sign a data use agreement (DUA) and/or material transfer agreement (MTA). These agreements serve as contracts that govern the transfer of data between the data provider and the data user (either an institution in the case of MTA or an individual in the case of DUA). For MTAs, most higher education institutions have a dedicated office (often called a technology transfer or intellectual property office) that needs to be informed and will help researchers navigate agreements. Both MTAs and DUAs specify the conditions under which data may be used, potential restrictions, and requirements for data storage, reporting, sharing, etc. Once access to the data has been granted, it is the responsibility of the researcher to comply with the requirements specified in these agreements. As such, it is very important to regularly revisit the agreement and ensure compliance and readiness for audits.

#### Determine whether ethical approval is required

The acquisition of data from human subjects requires ethical and regulatory oversight through formal approval by an institutional review board (IRB) or ethics committee. All big data resources have such ethical approval at the acquisition site(s), but they vary in terms of the need to obtain additional local ethical approval for secondary data use. Some datasets (such as the Human Connectome Project) consider the combination of variables as sensitive information despite the removal of names, addresses, dates of birth and other explicit identifiers, and therefore require local ethical approval (or a formal exemption decision) for all data users. Other datasets (such as the UK Biobank) consider the dataset anonymized and do not require local ethical approval for data users.

#### Maintain oversight over approved data users

In general, data may only be shared and used by individuals who are explicitly named under a DUA, MTA, and/or IRB/ethical approval. This sounds easy enough, but can rapidly get complex when considering growing laboratories and collaborations between multiple laboratories and when using shared high performance computing systems and/or cloud computing services. It is important to regularly review user lists to remove people who have left the team and add new students and collaborators. In high performance computing or cloud environments, careful file permission management and review is important, and “root” users outside of researchers (e.g., facility staff and leadership) may need to be included in DUA/MTA/IRB user lists to reflect their data access.

#### Acknowledge data resources in talks, abstracts, and publications

Creating and sharing a dataset is a substantial amount of work and one of the most important aspects of MTA/DUA compliance is therefore to ensure that the data resource is appropriately acknowledged in resulting abstracts, talks, and papers. What “appropriate acknowledgement” entails varies between data resources. Some datasets require an acknowledgement statement that includes the application ID, whereas other datasets may require citation of specific paper(s) or co-authorship. Explicit instructions for acknowledging a resource can usually be found in the MTA/DUA and/or data resource website.

In summary, using big data involves a contract that specifies the rules and requirements of data usage. Users of big data resources all carry the responsibility to familiarize themselves with the rules and ensure compliance for themselves, and their students, collaborators, and peers.

### Recommendation 2: Planning the analytical study design

When researchers conceive of a study that involves new data acquisition, this necessitates extensive planning and decision making to determine the sample size, inclusion/exclusion criteria, procedure, experimental conditions, measurement instruments, etc. With big data, there is a temptation to skip or shortcut the planning stages and jump straight in. Even though all the data already exist, the most robust and impactful uses of big data require similarly extensive planning and decision making before the start of a project.

#### Define a specific research question

Secondary data analysis of existing big datasets is often equated with exploratory (rather than explanatory or hypothesis-driven) research. However, it is very much possible – and even common – to perform hypothesis-driven research using existing data. Big data resources typically measure a very wide range of variables in a large (population) sample, and are therefore likely to contain relevant variables and cohorts to test very specific hypotheses and research questions. As such, one of the most important suggestions when using big data is to identify a specific and well-defined research question and set of falsifiable hypotheses prior to data analysis.

#### Plan and preregister the project

Big datasets (especially epidemiological cohorts) can be used to address an almost infinite number of specific and well-defined research questions. However, each of the possible research questions may require a different subset of the available variables, a different subset of the total number of participants, and a different analytical/statistical approach. Preregistration is one of the most helpful tools to encourage careful planning and decision making prior to the start of the project [35]. Preregistration is the practice of submitting a detailed study plan to a time-stamped repository (e.g., osf.io or clinicaltrials.gov), which enhances transparency, limits researcher degrees of freedom, and reduces publication bias [36, 37]. Study plans can either be made publicly available or can be kept embargoed until study completion to protect plans and ideas. Although preregistration was originally adopted for new data acquisition, the practice is equally valuable for secondary data analysis studies [38, 39], and the Open Science Framework offers a dedicated template for secondary data preregistration (https://help.osf.io/article/229-select-a-registration-template). Building on the concept of preregistration, a registered report is a preregistration (often consisting of an introduction and methods section) that is submitted for peer review to either a specific journal or a peer community registered reports consortium, in addition to being submitted to a time-stamped repository [40]. Once the “stage 1” registered report has been accepted, a journal commits to publishing the “stage 2” manuscript upon completion of the study regardless of the study findings. As such, a registered report determines the publishability of a study based on its hypotheses and design, instead of the findings. In summary, both preregistrations and registered reports are useful tools to encourage careful planning.

#### Determine the sample criteria and size

An important question to address in study planning is to determine the criteria for the sample. Depending on the research question, it may be beneficial to enforce post hoc inclusion/exclusion criteria to select sub-cohorts from big data for focused analyses on individuals with specific conditions and symptoms. When starting from an epidemiological dataset such as the UK Biobank, it is often feasible to retain relatively large samples of hundreds or thousands of participants even after enforcing additional inclusion/exclusion criteria. Missing data are another important aspect to look out for when determining the sample criteria, as the sample size may reduce substantially when only including participants with complete data on all relevant variables.

#### Plan for replication

If the sample of participants that meet inclusion/exclusion criteria and have complete data is sufficiently large, it may be possible and desirable to split the data into exploratory and confirmatory samples. Here, statistical model parameters are estimated in the exploratory sample and applied (without re-fitting) to the confirmatory sample to determine the effect size in a held-out sample. Beyond splitting the sample, another opportunity of the availability of several big data resources is to consider repeating the analysis in an entirely separate data resource (that may have different imaging parameters, clinical instruments, or demographic distribution) to perform an even stricter test of generalizability as opposed to replicability/reproducibility.

#### Validate instruments

Although big datasets often measure many different constructs, the desired breadth of information can sometimes come at the cost of the depth or quality of the adopted instruments, especially in epidemiological study designs such as the UK Biobank. As such, clinical instruments that are shortened or not yet fully validated may be adopted. Although data users do not have control over the instruments measured in a big data resource, it is possible to perform separate research to rigorously validate non-standard measures where needed. For example, the four-item recent depressive symptom (RDS) scale [41] and cognitive test battery [42] were previously validated for the UK Biobank.

#### Prepare and inspect data

Once a research question has been set and preregistered, the sample and replication plan has been identified, and instruments have been validated, there is one step left to consider before moving forward with data analysis. To avoid unexpected findings and obstacles down the line, it is useful to look at the data by plotting and inspecting data distributions. This step enables researchers to identify and investigate outliers, and can help uncover response coding challenges such as inconsistent missing data codes (e.g., 999, NaN, −7, etc.) or inconsistent coding directions (e.g., going from least to most or vice versa).

In summary, impactful use of big data requires extensive planning before the start of a project. Preregistration offers a useful tool to encourage thoughtful planning and helps avoid the temptation to jump straight into data exploration.

### Recommendation 3: Consider pooling data with diverse study designs

Despite the immense utility of large scale existing public brain imaging databases (see a selection in Supplementary Table 1), there are times where such data sources have relatively little or no information on a specific topic of interest. Existing collections, by design, generally focus on population samples, recruiting largely healthier populations. In fact, the UK Biobank participants are on average, healthier than the general population, suggesting a healthy volunteer bias [43]. Furthermore, data collected from more clinical populations can contain more sensitive information that may make it not as readily, or publicly, accessible. Some researchers may only be able to share data from “control” subpopulations of their study publicly. If a research group is interested in a specific clinical condition, or for example, details into treatment history and responses, or specifics into disease subtypes and progressions, they may have collected such data as part of a single center study. Thus, when looking to increase such sample sizes, it may require collaborations with other researchers interested in the same question as opposed to, or in addition to, searching the public domains. This work can generally be done in either a centralized or federated manner.

#### Differences between centralized vs. federated repositories (Fig. 1)

A centralized repository is a single centralized storage location, such as the NDA, for all of the data collected by any number of smaller organizations. Under ideal circumstances, centralization may lead to increased data integrity and transparency, by enforcing common data models (CDMs) and scientifically meaningful validation rules, by removing duplication, and by streamlining access to resources. Centralization is one solution to growing concern over the lack of reproducibility of publicly funded studies in an increasingly collaborative big-data environment. Federated repositories, in turn, are collections of summaries or metadata about data that are stored in different places. Under ideal circumstances, they reduce the need for agreement on a fixed and existing data model or structure, distribute the cost of storage, and allow for (meta) data sharing where consent or embargo prohibit centralization. Federated approaches often leverage linked meta data structure via RDF (Resource Description Framework) and OWL (web-ontology language), which make searchability much more powerful over traditional relational database designs. Intuitively, the semantic web tools can be understood as the power of googling for publications vs. constructing a PubMed search; in practice, it is very difficult to incentivize the use of standards in annotation capture. Moreover, for federated approaches, standardization is lacking and the tools with which to work with federated data are under development [44].

**Fig. 1:**
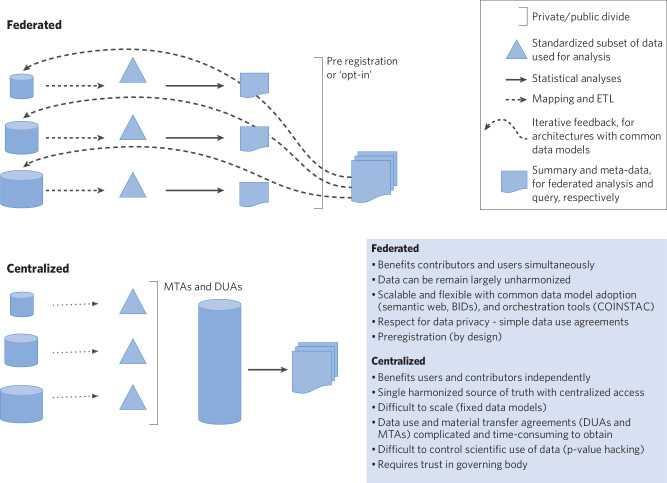
Overview of differences between Federated and Centralized Data Resources. Acronyms: BIDS (Brain Imaging Data Structure; https://bids.neuroimaging.io/), COINSTAC (Collaborative Informatics and Neuroimaging Suite Toolkit for Anonymized Computation) [132], ETL (extract, transform, and load operations), MTA (material transfer agreement), DUA (data use agreement).

#### Getting involved with consortia

Many coordinated collaborations have taken hold in the form of large-scale consortia to overcome issues with small, single source datasets, in an effort to enhance reproducibility and reliability in neuroimaging and psychiatry. Some consortia are created to collect data across multiple sites with a prospective design in a harmonized and standardized manner. Other consortia such as the Enhancing Neuro Imaging Genetics through Meta Analysis (ENIGMA) Consortium [30], were designed to work with researchers in a coordinated effort to pool data retrospectively across existing studies. Here, we briefly discuss getting involved with ENIGMA in particular, as the ENIGMA infrastructure and design allows researchers to participate in specific projects based on data availability and interest.

The ENIGMA Consortium is composed of many working groups. The majority of the working groups are dedicated to the study of specific neurobiological sources of variation, whether in clinical neuropsychiatric populations or the more general population. Working groups are often initiated when a researcher and several colleagues realize the need to pool their datasets and resources together, and established consortia such as ENIGMA provide the infrastructure for sustainable growth. Within each clinical working group, an initial project generally assesses the brain differences in individuals with a particular diagnosis, compared to unaffected controls. This initial project requires data curation and standardization (imaging and clinical) and highlights the compatibility across datasets. Once such initial efforts are in place, more specific projects can be proposed by any working group member. These projects often target more specific aspects of the condition, such as the effect of medication on the brain, rates of brain structural change over time, and how different symptom profiles may contribute to different brain network differences. Each working group member may choose to get involved in a particular project, which is often the case when they have relevant data available, or not. Membership into the ENIGMA working groups is not limited and interested individuals are encouraged to reach out and get involved. This makes it feasible for individuals with smaller single-site datasets focused on clinical neuropsychiatric populations to become part of a network of like-minded individuals and collectively improve power and reliability in brain imaging studies. Pooling diverse sources of data, especially those that were originally conceived and collected independently, is not trivial. We discuss some challenges and current areas of active research in section Looking Ahead: Opportunities and Future Research Directions.

### Recommendation 4: Planning for data storage and computing

#### Identify computing resources

As a result of the large size of big data, an important challenge for new and existing users is the substantial storage, computing, and data management requirements to maintain and run analyses on a big dataset. A variety of resources and approaches are now available to address this challenge. The first option is to leverage local high performance computing resources. The majority of research-oriented higher education institutions offer some centralized high performance computing resources, so a search for such local resources may be a suitable first step. An alternative to local high-performance computing resources is to use cloud computing facilities such as Amazon Web Services (AWS), Microsoft Azure, Google Cloud Platform, IBM Cloud, Oracle Cloud, DGX Cloud, Huawei Cloud, Alibaba Cloud, and DigitalOcean. Increasingly, big data and neuroimaging resources offer their own cloud presence, for example the Human Connectome Project dataset is available as an AWS “bucket,” the UK Biobank dataset is available in its Research Analysis Portal powered by AWS, and NITRC offers their own computational environment [45]. Institutions and funders are also increasingly offering support for cloud-based research, including NIH’s STRIDES initiative, NSF’s ACCESS program, and the NIMH Data Archive (NDA). A final alternative is to distribute the burden of analysis across sites/studies using a federated analysis approach [44], and subsequently collating the results in a second-level meta or mega analysis. This distributed analysis alternative to data sharing has been very successfully employed in ENIGMA working groups [46, 47]. Beyond the primary storage and computing environment, big data have increased the need for automated tools to organize, process, and share neuroimaging data. Please see Box 2 for a select overview of such tools.

#### Understand the demands of secondary analysis of big data research

Beyond storage and computing resources, effective use of big data requires substantial personnel time and effort. The bigger the dataset, the more time is involved for (i) downloading, organizing and maintaining datasets, (ii) preprocessing, cleaning, and harmonizing datasets, (iii) implementing and running analyses in high performance computing and/or cloud environments, (iv) code reviews, replications, and other robustness checks, and (v) data sharing of outcomes and results, not to mention the time spent (vi) training users on relevant tools, data intricacies, and computing resources. The realities of these time consuming aspects of big data research are increasingly shaping the educational trajectory and job opportunities of students, staff, and faculty.

In summary, careful use of big data research requires substantial training, personnel, and computing resources, even though the data have already been acquired. Institutions and funders are responding to these demands by offering training and computing resources. Nevertheless, continued awareness of the time and cost required to perform robust big data research amongst peer researchers, paper and grant reviewers, institutional hiring and tenure committees, and funders remains critical to encourage optimal big data usage.

Box 2 Resources for big data research in neuropsychiatry

**Table:** 

Topic/ Name	Description	Link/ reference
BOUTIQUES	Integrate and execute command-line applications across computational platforms	https://github.com/boutiques
BIDS	A simple and intuitive way to organize and describe your neuroimaging and behavioral data	https://bids.neuroimaging.io/
BALSA	Data & results sharing site based on workbench view	https://balsa.wustl.edu/
NITRC	NeuroImaging Tools & Resources Collaboratory including data sharing and computational environment	https://www.nitrc.org/
OpenNeuro	A free and open platform for validating and sharing BIDS-compliant MRI, PET, MEG, EEG, and iEEG data	https://openneuro.org/
The Good Research Code Handbook	Teach how to organize code so that it is easy to understand and works reliably	https://goodresearch.dev/
Open Science Framework (OSF)	Flexible repository to share research ideas, preregister a study, store collected data, etc.	https://osf.io/
Preregistration	The Open Science Framework offers a dedicated template for preregistration of secondary data analysis projects (named ‘secondary data preregistration).	[38, 39] https://help.osf.io/article/229-select-a-registration-template
DataLad/ BABS	A reproducible and generalizable software workflow for analysis of large-scale neuroimaging data collections using BIDS Apps	[142, 143]
NDA (NIMH Data Archive)	Infrastructure for sharing research data, tools, methods, and analyses enabling collaborative science and discovery	https://nda.nih.gov/
DANDI	The BRAIN Initiative archive for publishing and sharing neurophysiology data including electrophysiology, optophysiology, and behavioral time-series, and images from immunostaining experiments	https://dandiarchive.org/
IDA	The image data archive for securely distributing neuroimaging data alongside other datatypes. Used to distribute data from the Alzheimer’s Disease Neuroimaging Initiative, and many other public and private/investigator-directed data repositories	https://ida.loni.usc.edu/

### Recommendation 5: Be aware of pitfalls

#### Report effect sizes in addition to significance

The flipside of having a large amount of statistical power is that relatively small effects can be highly statistically significant [48, 49]. As discussed in the introduction, small observed effect sizes do not necessarily imply that the underlying association (free of measurement noise) is unimportant. Nevertheless, reporting and consideration of effect sizes – in addition to significance – is increasingly important to ensure transparency and aid comparisons between studies with potentially vastly different sample sizes.

#### Be mindful of confounds

Another flipside of having a large amount of statistical power is that sensitivity to undesirable effects such as the role of confounding variables is also greatly increased [48]. As such, careful confound control is increasingly important in big data analyses [50]. One option to address confounds is to subsample from the large dataset such that groups are matched for confounding variables, for example by using propensity scores to ensure that “patient” and “control” groups do not differ on potentially confounding dimensions such as age and sex [51].

#### Implement machine learning algorithms responsibly

The riches of big data create opportunities to use complex analytical models including machine learning and deep learning. Prior work has established important best practice guidelines for such models [52–56]. One particular pitfall is leakage in cross-validation which occurs when information from test participants is inadvertently used to inform model training. Such leakage can occur due to participant relatedness (e.g., siblings/twins cannot be treated as independent subjects) [57] or confound processing or other group-level steps performed prior to machine learning (e.g., regressions performed across all subjects expose and potentially bias training data by using information from the full dataset) [58, 59]. Such leakage is avoidable by implementing processing steps as part of the cross-validation folds and designing cross-validation splits carefully. The robustness of findings can furthermore be enhanced by estimating model accuracy across multiple repeated cross validation folds to assess variance (also known as shuffle splits, cross-validation bagging, or resample aggregating) [60]. Importantly, out-of-sample prediction using a truly independent dataset is important in addition to partitioning one dataset through cross validation to assess realistic model performance on prospective data (e.g., newly acquired patients) [61]. A second pitfall is model bias due to non-representative samples [62], which is more challenging to avoid altogether, although replication in additional datasets can help assess bias.

## Acquiring or contributing to new big data

### Recommendation 1: Consider the participants first

The neuroimaging data being discussed in this review are ultimately from human participants, and while it is often shared and distributed in the form of spreadsheets, the human element is critical to every aspect of study design and even the interpretation of findings. It is important to keep the participants in mind, especially when setting out to collect personal information and health related measurements and assessments. Not doing so may hamper recruitment efforts and make retention of participants particularly challenging; it may also bias the participant pool and research findings. Here, we list several considerations that may help ensure successful recruitment of participants.

#### Working with your IRB

As with all biomedical research involving human subjects, it is imperative that your institutional review board (IRB) or ethics committee review the study design, the recruitment strategies, and the resource sharing plans. These professionals are trained to review and monitor biomedical research to help ensure the protection of the participants. In doing so, they can provide many good suggestions and ideas to minimize participant inconvenience or discomfort, and maximize recruitment efforts, especially for patient populations. It may be advisable to work closely with your designated IRB/ ethics representative to discuss study aims and maximize successful recruitment and retention. It may be beneficial to contact your IRB/ ethics committee early on to get valuable insights while designing your study.

#### Providing clear details on study risks and benefits, consent to share data, and a way to withdraw

Many variables can be combined to identify individuals even in anonymized datasets, and it is important that participants be aware of potential risks and what the research team is doing to minimize risks. The benefits of data sharing are also important to share with individual participants [63]. Participant consent forms should have specifics to ensure the participant’s consent to data sharing beyond the investigative team, and ways to withdraw from the study for future assessments and remove the shared data from the public domain. This should include providing a specific email address and contact person for the participant to contact should they wish to be removed from the study. This would also necessitate collecting email addresses and contact information from researchers using the data in efforts to communicate study withdrawals and request data removal; this may also be accompanied by requiring researchers to renew their data access regularly to ensure they are working with the latest set of participant data. Without these safeguards, the population may inherently be biased towards individuals who are generally well represented and overall healthy, i.e., individuals who are less concerned about their health information being widely accessible. By allowing individuals to clearly decide how their data may be used moving forward, and what aspects of the information they provide they are comfortable to share more broadly, the efforts to ensure participant safety may therefore simultaneously improve recruitment efforts for underrepresented and clinically sensitive populations.

#### Working with a broad range of populations

There are several ethical and practical challenges in conducting mental health research in human subjects, and important procedures to follow, particularly when conducting multi-ethnic or international research. For reviews, please see [64–66]. While striving to make your research data as openly accessible as possible is an important goal, it may not be an immediate possibility if it causes discomfort and distrust to your participants. However, there are alternatives to completely open data, and other opportunities by which to participate in big data research with your study. This may include for example, only making a subset of data available, whether it be a subset of participants (for example, the controls in a case/control design), or a subset of collected variables (for example, imaging derived features, but not genetic data). When working with a new population of individuals, it may be advisable to determine in a pilot study and/or consult with stakeholder advisory committees/boards (e.g., individuals with lived experience, family members, patient advocates, etc.), whether there may be information related to specific questions or analyses with which the participants are not comfortable. Ensuring that such questions and analyses are removed from data use agreements may establish improved trust and communication between the participants and the researchers. For example, one long-running multigenerational study funded by the National Heart Lung and Blood Institute (NHLBI), the Framingham Heart Study (FHS), which started in 1948, laid out clear areas of study in its consent form to participants. For example, as seen in Supplementary Fig. 1, from one version of the FHS consent forms (Available: https://www.framinghamheartstudy.org/files/2017/08/Omni-Gen-1-Cohort-Exam-3.pdf), participants are asked to strictly agree to whether they want their data used for the original goals of the study (heart disease, lung and blood disease, stroke and memory loss). The consent form establishes a separate consent for using data related mental health conditions, including depression and alcohol use. Older versions of the form did not ask for this separate distinction, so individuals consenting on original versions of the form arguably did not directly agree to data use beyond that of the original goals of this study. Data use must be obliged by the consent of the participants.

### Recommendation 2: Detail your study design and plans for potential study expansion from the start

While designing your study, it is, of course, necessary to think of optimizing your scientific agenda and the appropriate aims to answer key gaps in our knowledge. While the aims of the current proposal are the most critical, thinking ahead beyond these aims will ensure the study gains visibility and momentum such that it is eventually allowed to grow. So in designing or describing your study, it may be beneficial to collect or document a few more items than you may have been considering for the purposes of your specific study, and to perhaps do so in a structured manner. This may prepare you and your study for better integration with existing and future research. Some considerations are listed below, although this is only a partial list.

#### Study design, participant inclusion/exclusion criteria, and recruitment strategy

Making detailed descriptions of inclusion and exclusion criteria for all participant groups, whether population based, or clinical cases and controls is important for comparing different datasets, and drawing comparisons between them. Even when examining individuals diagnosed with the same condition, individuals on or off medication may have differences in brain structure, as was found in work on obsessive compulsive disorder [67]. The severity of the condition may also have notable effects on brain structure, and if recruitment focuses on different subsamples of a population, that would be important to highlight; for example very distinct patterns of brain differences are seen in early stage Parkinson disease (PD) compared to controls than later stage PD compared to controls [68]. It is also important to document recruitment strategies, as for example, a control group recruited from a college campus may have very different demographics than a control group recruited from the general population. Understanding these differences in clinical populations as well as the general population, or “controls” may help better formulate recruitment strategies and understand, or minimize, biases in the target sample. Detailed descriptions of such criteria and strategy, in addition to whether data are exclusively cross-sectional, or any parts will be longitudinal, and of course sample size, can inform future users of your dataset and collaborators as to how your dataset may complement their study and add to their research goals.

#### Collecting socio-economic and demographic information

Going beyond the minimum and collecting a few relatively quick to obtain details on participant socio-economic and demographic information may open opportunities to be involved in more research than originally anticipated. It is always important, and often required, to capture demographic variables such as age and sex as federally defined for recruitment monitoring of funded initiatives. These often follow specific definitions, yet it may also be advisable to go beyond census options to provide an expanded list of possible gender identities and race/ethnicity options, and allow for self-identification. The federal and national definitions of demographic variables such as race and ethnicity allow an easy way to standardize these variables across datasets. However, these are often minimum categories and may be expanded, so adding options for self-identification may improve standardization in the future and allow your data to provide information on currently uncategorized and underrepresented individuals. For example on March 28, 2024, the Office of Management and Budget (OMB) of the United States officially revised their earlier recommendations to add Middle Eastern or North African (MENA) as a new minimum category in race/and or ethnicity categories when maintaining, collecting and presenting federal data on race and ethnicity [69]. This was the first change to the OMB minimum categories since 1997. If data collected in the US before this date did not include MENA as an option, there would be no way to tease apart who in the cohort may identify as part of this newly formalized ethnic group. Representation across a variety of sociological, economical, demographic and various aspects of personal background and identification may allow you to expand your research aims and assess additional correlations beyond those of the original study. While the diversity of representation in your study across all these aspects will be dependent on the overall goals and aims of the research, and whether it is meant to be targeting a more focused population or a more heterogeneous one, capturing details beyond the bare minimum will help ensure data re-use and continued evaluation. For example, going beyond socio-economic status and/or area deprivation index to also obtain geocodes related to the participants’ living environment, may allow for future work relating collected brain health variables to regional exposures and specific pollutants [70].

#### Consider taking part in collaborations/consortia early on

After clearly documenting the research goals and preparing the study design, it may be advisable to get in touch with potential collaborators and consortia early on. Are there aspects of the design that can be easily combined with those of other researchers? Are there aspects of the design that may benefit from modifications to add to increasing the sample size for what already exists? What important questions are not currently being addressed by consortia due to lack of data, and can your study help gather the data and resources to answer these questions?

### Recommendation 3: Plan for FAIR data sharing from the start

When planning to share data, it is important to ensure that the resource is findable, accessible, interoperable, and reusable, or FAIR [71] while also following the criteria described above to ensure participant safety and comfort. Although these may change throughout the course of the study, we provide a few considerations and recommendations as to what to share, when to share, how to share and where to share.

#### With whom to share?

The sharing of data does not have to be uniform across all potential researchers. Many existing repositories provide different tiers of access, with some data being more restricted than others, with more safeguards in place, including material transfer agreements with the researcher’s institution. Other data may be less sensitive, or modified to be as such, and can be made publicly available. For example, while knowing the sex and exact age and family structure of an individual participant may require some restricted access, providing information on participant sex and a five-year windowed age bin without information on family structure, may not contain any sensitive information and may have unrestricted access. This, for example, is how data from the Human Connectome Project may be released. Regardless of what data are released and with what level of access, it is always important to obtain at least a contact email from the user, such that any updates in data availability, data quality, and error identification can be communicated to users of the resource.

#### What and when to share?

The what and when to share data go hand in hand. We discuss three possible time courses for sharing data, and potential types of imaging data that can be shared, although a similar framework would apply to other datatypes.

##### Throughout the course of data collection

Especially if the study is collecting data from a large number of subjects, or longitudinally, it may be preferred to begin sharing data in waves, and continue to have scheduled data releases as data continues to be collected. Providing access to the raw or minimally processed data may be preferable at this initial stage. Minimally processed data may include defacing T1-weighted (T1w) scans, which can help prevent re-identification [72], and preprocessing multivolume echo planar magnetic resonance imaging (MRI) such as diffusion and functional MRI scans to remove motion and distortion artifacts. It is also beneficial to assess image quality as data is being collected. Quality assurance throughout the process of data collection and distribution may avoid downstream analysis on images with excessive artifacts. While providing imaging derived traits from fully processed images is also advisable, it may be required to process data many times until the end of the data collection due to novel tools and pipelines that become available throughout the course of the study. In all cases, it would be important to fully document every processing step and any variations in processing version. Any differences in data acquisition that occurred throughout the course of the study and data availability should also be noted. While making data available as it is collected allows more researchers immediate access to the data, it may also come at the cost of multiple batches of processing and undesired sources of variance. It may also be possible that data quality issues, particularly those assessed via the use of statistical outliers, are not identified in smaller batches.

##### After data collection

While the same raw and minimally processed data types may be shared after data collection, it may also be in the researchers interest to also provide derived imaging measures once data collection is complete such that all data processing occurs only once. One advantage of making data available after data collection is complete is that only one wave of initial data release is needed, which includes a single processing batch. However, this comes at the cost of delayed access to the data for many interested investigators.

##### After study/grant termination

Some investigators may choose to further delay the sharing of data until after the completion of the study entirely, assuming the study calls for testing particular hypotheses with the data. This would allow the investigative team additional time and motivation to process the data, derive biologically meaningful imaging traits and associations, and possibly more importantly, ensure data have been vetted and quality controlled. One drawback of this is that there may be limited resources available after the fact if issues were to be identified with the data after the investigative team is no longer funded to support the project and issue new data releases. It is important to ensure the data is thus stored on a platform that allows for community engagement and updates.

As mentioned earlier, it may be advisable to share data according to various administrative permissions. For example, while the face on a T1w image may be used to identify an individual from a database [72], de-facing MRI scans may also have adverse consequences for downstream data processing methods that use the full brain image, such as brain age prediction [73]. Added safeguards can be put in place when investigators request original data.

#### How to share?

In section Using Existing Big Data - Recommendation 4: Planning for data storage and computing, we list resources that are also applicable to sharing imaging and non-imaging data, computational resources, and study design frameworks and research plans. Ensuring a sustainable framework for data sharing will ensure the longevity of the study. It is important that researchers who are obliged to share data are able to do so, and not simply appear to share data [63].

### Recommendation 4: Standardize, annotate, monitor, support

#### Prioritize deep or wide data collection responsibly

Longitudinal epidemiological data provides an opportunity to quantify and compare the reliability and accuracy of clinical and cognitive instruments by repeated collection over time. However, the benefit of deeply phenotyping measures or domains of interest in epidemiological data must be weighed against the need for coverage, with the former having better potential to make strong statements about specific associations and measurement utility in a specific domain, and the latter better poised to capture major trends across domains. Balancing deep vs wide phenotyping approaches is especially important for human subjects, where time and effort place limitations on the number of measures that can be collected. Regardless of variable choice, it is preferable to avoid reducing continuous forms of variables to categorical derivatives, such as body mass index (BMI) to “obesity” (yes/no). Moreover, when publishing data, consider including item level responses in addition to summary scores so that new combinations of variables can be constructed.

#### Harmonize instruments, versions, collection devices, database schema, and variable names

Where data are pooled for analysis, the task of harmonizing clinical and cognitive instruments can cripple productivity with endless variable mappings and ETL operations (extract/download data from sources, transform/reformat/ massage into new formats, and load/merge/concatenate result with larger dataset). Even instruments based on the same publication may suffer from insufficient labels, unit changes, or a lack of consistent or intuitive naming conventions. Schematic decisions, such as whether data are wide (one row per subject), tall (one row per variable), and/or longitudinal (multiple rows per person) can also place a considerable burden on the time it takes to pool information across multiple studies. Moreover, instruments may be grouped into larger collections by file size, clinical domain, or permissions group, according to the priority of the individuals(s) tasked with organization. Insufficient information with which to locate instruments of interest within such groupings add to downstream harmonization time. For these reasons investigators should consider using curated and publicly available instruments wherever possible, such as the National Institute of Health (NIH) Toolbox (Ipad Application, normal export format; [74–76]) and the National Cancer Institute Automated Self-administered 24-Hour Recall (ASA24) [77], and plan for the personnel needed for clinical/behavioral harmonization. We estimate that on average, it would take a full time employee one month to identify the targets and transformations necessary to concatenate 1000 variables in one dataset with 1000 variables in another (assuming a 1:1 mapping exists), *after which* all operations required for data merging can be encoded for reproducibility.

#### Augment data dictionaries with links to literature

A data dictionary describes the names, definitions, and attributes about all data elements that are captured as part of a research project both at the level of instruments (i.e., a summary score for an entire survey) and variables (i.e., each item within a survey). Dictionaries from one study or instrument need augmentation and standardization before they can be concatenated with other data dictionaries or linked to other study datasets via a “Crosswalk.” A crosswalk is a data dictionary with extra information about any ETL operations required to map data from one study into a public archive (or other study); in many cases it is simply a linking of variable names from source to destination.

Dictionaries about instruments (Supplementary Box 1) should be created by investigators when designing the experiments and data monitoring plans; they should not be assembled after data collection, when institutional knowledge about their content has dispersed with personnel. Such dictionaries should include the published name for collected instruments and its grouping (domain, module, construct, etc), short names or aliases, a citation, and any other context that would distinguish collected data from another similarly annotated measure (version or collection platform), and any in-house changes to the published instrument on which it is based, such as omitting or adding questions. Dictionaries of variables (Supplementary Box 2) must be linked to the instrument dictionary (e.g. by shortname). Wherever possible, variable names should employ a naming convention that concisely alludes to content, question order, and grouping. However, any changes made to variable names output by the platform on which data were collected during the course of ETL operations must be rigorously tracked so that processing can be reproduced with the arrival of new raw data. Special care should be taken when labeling variables that have been answered by one person on behalf of another (e.g., parents about children). Data collection events for longitudinal data, and skip or branching logic such as those available in event maps and data dictionaries in Research Electronic Data Capture (REDCap) [78, 79] projects should not be omitted from collections of supporting documentation if they are available, as such logic informs patterns of missingness and participant fatigue. Lastly, dictionaries should detail the set of all possible value/label pairings, missing codes, and units for all variables.

#### Include persistent identifiers and/or variable mappings to public repositories

Ideally, both the instrument names and the variables within these instruments would be linked to unique and persistent identifiers [80–82]. Unique and persistent identifiers, such as those curated with vocabularies and ontologies in the Neuroscience Information Framework (NIF; [83]), are long-lasting links to unchanging, and ontologically informed definitions and concepts. Such identifiers are unchanging persistent links to specific versions of publications or datasets, much like digital object identifiers (DOIs). The practice of associating every item in a data dictionary with a persistent identifier ensures machine-readability and prevents drift of meaning during repeated mapping processes. If ontological identifiers are unavailable, and the dictionary to which one is mapping is fixed, such as the NDA, it is critical to maintain a crosswalk between local and remote annotation by way of variable maps, so that users having questions from data downloaded from the public archive (with non-standardized and often non-informative labeling) can be directed to more informative annotation collected at your institution, without the need for reverse harmonization.

#### Expect delays

Submitting data to, and downloading data from centralized public archives requires administrative support, domain knowledge, and agility using the command line and programming languages to interact with custom Application Programming Interfaces (APIs). Some repositories have strict limits on volumes of download (e.g. 20Tb per month at the NDA). Data use agreements can be extensive and require signatures at multiple levels of a research institution’s administrative hierarchy. Repository support can be minimal, undocumented, and/or inconsistent. Often, the steps required to acquire or submit data can take longer than the analysis planned for the data.

#### Broaden data cleaning and monitoring plans

Data monitoring plans for big-data studies need to include realistic estimates of the person-time required for storage, compute, download, upload, and analysis, in addition to the more traditional budget line-items [84]. Neglecting to include well-established rigorous data cleaning traditions [85] or omitting new layers of meta-cleaning will result in measurable signal loss and should therefore be avoided. Many recommendations for data cleaning include checks for outliers and missing data (structured or unstructured), as well as advice on how to prevent typos in key identifiers and methods of creating validation rules. Data at large scales requires another level of cleaning and management, such as having a mechanism in place before data collection begins to regularly report on numbers and distributions from data types and collection sites. This way systematic issues with infrastructure or protocol deviations can be identified and addressed in real time. To achieve efficient problem solving, regular communication between data managers, project managers, coordinators, information technology (IT) staff, and scientists should be prioritized. The breadth of expertise required for various tasks can easily result in decisions made without regard to scientific relevance. Without scientific stewardship over the process of data collection and extraction, much of the effort to collect data may proceed in vain.

#### Plan for ongoing support of collected data

Well-managed, rigorously collected big-data will see widespread use; during this time, issues will be found, and questions will be asked. Without a plan for supporting a growing community of users, data will be as good as the annotation of the last person with institutional knowledge about its scientific content or collection mechanism. The scope of support and designated point persons for live and completed collection should be identified and documented a priori from activities such as fielding questions from users about mechanics and/or scientific meaning of various data components, extending documentation, curating and patching known issues, re-releasing data, and managing a website.

### Recommendation 5: Combining multimodal neuroimaging and other data types

#### Adopted multiple imaging modalities with harmonized acquisition parameters

The majority of neuroimaging studies already include a range of imaging sequences. Different imaging sequences offer complimentary measurement types (e.g., functional, structural, diffusion imaging), and some sequences may offer greater sensitivity to clinically relevant features (e.g., T2-weighted imaging, susceptibility weighted imaging). In addition, the adoption of recent advances in functional MRI sequences that offer improved signal quality (e.g., multi-echo multi-band sequences; [86]) and other practices that improve signal to noise ration including sufficiently long scan durations [87]. Although most big datasets include neuroimaging of all modalities (e.g., structural, functional, diffusion), prospective harmonization of sequences is lacking. For example, extensive imaging differences are present even amongst the “connectomes related to human disease” studies that were inspired by the human connectome project [88]. Although strict harmonization is not feasible due to differences between scanner make/ model and the need to balance the desire to adopt novel sequence improvements versus maintaining backward compatibility in large and/or longitudinal studies, it is beneficial to match sequences to other existing datasets to encourage replicability and support harmonization.

#### Include complementary data modalities

Neuroimaging data can be greatly enhanced by leveraging genomics, epigenomics, transcriptomics, proteomics, metabolomics, and additional markers identified from blood, saliva, plasma, urine, skin, pupillometry, pulse oximetry, respiratory bellows, and wearable technologies [89–91]. In addition to the value of omics data, samples that are enriched for genetic relatedness (e.g., twin/sibling enrichment adopted in studies such as HCP-Young Adult and ABCD) offer additional research opportunities. In addition, mobile health (also known as M-health or Ecological Momentary Assessment; EMA) can be used to regularly probe participants to self-report (e.g., regarding mood, attentional focus, diet, etc.), or to participate in gamified cognitive tasks and healthcare interventions [92]. Lastly, passive sensing data can include social media information and passive mobile data such as global positioning system and linguistic information from text messages [93], although ethical considerations such as data protection and privacy should be emphasized [94].

#### Plan for integrated analysis

Although more (and longitudinally repeated) data types and sources offer many benefits such as holistic precision information and opportunities to assess and increase reliability, the vastness of data can also cause challenges. In particular, dealing with large numbers of variables creates challenges for analysis and interpretation. Integrated analytical approaches to date leverage dimensionality reduction [95], clustering [96], feature selection [97], machine learning [97], and shared latent spaces [98, 99]. Notably, the development of novel analytical approaches for high dimensional analyses is an active research field. In the presence of large numbers of variables, interpretability can suffer as a result of high dimensional results and/or complex analytical approaches. Again, the development of interpretable machine learning techniques is an active field of research [100, 101].

In summary, big datasets can be greatly enriched by the inclusion of varied and innovative data types including genetic and biological markers measured on the day of scanning as well as digital phenotyping. Although the richness of data variables can bring challenges for analytics and interpretation, integrated and interpretable analytics are a fast-moving field that will enhance the value of big data.

## Looking ahead: opportunities and future research directions

### Opportunity 1: Incorporating new data with legacy data

**Fig. 2:**
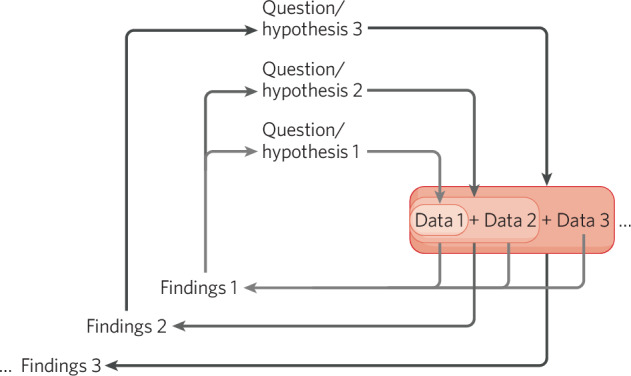
Continuous and virtuous research circles. From inside (lighter gray) to outside (darker gray), we show how adding more data can continuously add to research findings and lead to new research directions and opportunities, by allowing more and more questions to be asked. In this diagram, “Question/Hypothesis 1” is asked of “Data 1” to get “Findings 1.” As more data are added (Data 2, Data 3), this question can be asked continuously to update the findings. Findings 1 may then lead to novel questions asked in “Question/Hypothesis 2” that can be addressed with more data “Data 1+ Data 2,” while “Data 2” can also be used to address the first question, “Question/Hypothesis 1” alongside “Data 1.” “Finding 2” may then lead to a new “Question/Hypothesis 3” that can be answered only with the addition of a third dataset “Data 3” to the original two. Not all datasets need to be used to answer every question, for example “Data 3” in this case is not used for “Question/Hypothesis 2” to obtain “Findings 2”.

There are many exciting opportunities presented when data can be shared and combined with other data. For example, Fig. 2 highlights how one can not only gain statistical power in having more samples to address a particular question, but that such findings may drive new research designs and hypotheses to be tested. New and existing datasets can also be used to validate each other’s findings. However, as one may imagine, a major hurdle in pooling potentially disparate data is ensuring the compatibility of the data types. The variability in the image acquisition itself may contribute to differences in derived metrics, for example voxel size, number of gradient directions, number of repeats and many more parameters play a crucial role in the case of diffusion MRI [102], rendering completely different means and variances for the derived microstructure metrics. Statistical harmonization techniques can and have been routinely conducted to mitigate many of these “site-effects” [103–111].

When designing a new multisite study with new data collection, it would be advisable to maintain consistency in the set of data fields collected, and how they are collected, from diagnostic instruments to scanning acquisition protocols, which should be routinely calibrated through the use of phantoms. However, such prospective study designs are not always feasible, so retrospective post-hoc data pooling has become more common across multidisciplinary researchers. Consideration of measurement variability, such as site and scanner differences or differences in other forms of assessment tools, is an active area of research across many disciplines. Oftentimes, consensus, or lack thereof, can be determined through meta-analytical methods. Literature-based meta-analyses often combine results of published studies, which may have been conducted with different analytical and methodological approaches, and would downweight any null findings due to publication biases. More systematic coordinated meta-analyses, such as those conducted within several ENIGMA publications [112, 113], can be conducted in a federated manner, where the same analytical protocols are performed at individual sites on individual cohorts. without sharing individual participant level data. Next, the statistical findings from the individual cohorts can be combined statistically, often through fixed-effects or random-effects meta-analysis; this meta-analysis approach can be particularly useful for associations with potentially identifiable data types such as genome-wide genetic data [114]. Meta-analyses inherently take into account site differences and heterogeneity by weighting the effects of the individual sites by the inverse of the variance of the effect, which takes into account variance due to measurement error. On the other hand, working with centralized features from diverse datasets, or conducting “mega-analysis,” and analyzing all the data together, should incorporate methods to adjust for potential biases and variability due to methodological and acquisition based differences. While approaches often include adding fixed-effects dummy variables or modeling site as a random-effect, the need to perform “big data” analytics has driven a rapidly growing research area on multi-site data harmonization. Recent developments use batch-adjustment tools, such as ComBat [115], have had particular success in neuroimaging [108, 116]. ComBaT, related extensions, and similar statistical methods aim to harmonize derived features before running statistical tests of interest. Several works have empirically compared various harmonization strategies [46, 107–110, 117]. It is important to ensure that the harmonization approach chosen is appropriate for the data at hand, and to keep an eye on the growing set of resources and tools available. For example, for datasets that span a wide-age range, or incorporate other trends with known non-linear properties, methods that incorporate such terms, such as the generalized additive model in ComBat-GAM [106] may be appropriate. It is also important to ensure the data meet the assumptions of the harmonization approach. Overviews of several of these approaches, their limitations, future directions and more can be found in Refs. [103, 118].

Caution must be taken to ensure differences in population are not confounded with the perceived acquisition related differences. For example, a legacy dataset in older individuals acquired at 1.5 tesla being statistically harmonized with a newer dataset of adolescents, collected on a 3 tesla machine, may confound some variation in age group differences with the acquisition differences. In such cases, meta-analytic approaches where the replication of statistical findings from each dataset is assessed, may be valuable. It would be advisable to conduct leave-one-site out analyses to ensure one site, or dataset is not driving the overall effect, and when possible, to compare site specific trends even when the primary approach is a combined analysis. Artificial intelligence derived approaches such as those using style transfer based generative adversarial modals [119] aim to overcome such complexities by disentangling an image’s style from its content but as of 2024, require the full brain image as opposed to the derived traits, and are still an active area of research [103].

Even if the imaging acquisition remains the same, datasets may undergo variations of image processing pipelines. Inconsistencies in processing workflows are likely to create reproducibility and replicability issues [120]. Establishing how reliable each processing pipeline may be, and its compatibility with other pipelines, or versions of pipelines can help provide estimates as to what features in the image, or what areas of the brain, may be most susceptible to such workflow discrepancies [121]. Compatibility issues are also important to assess across non-imaging measures as well. When working across multiple studies, some of which have collected data in more than one way, it may be feasible to conduct large-scale compatibility studies from existing datasets, as in the case of assessments of suicidality across a wide range of neuropsychiatric risk assessment instruments [122]. Advanced data science approaches are also leading to novel approaches for identifying commonalities across different inventories [123]. As more data are being collected and shared, methods to standardize the processing and reporting of diverse datasets, including the data types and metadata within them, are leading to new developments in the area of multiscale high-dimensional data harmonization.

### Opportunity 2: Need for more reliable measures

The joint reliability of imaging and clinical measures places an upper bound on observable brain-symptom effect sizes [28, 29]. As such, there is an urgent need to improve the reliability of both imaging measures and clinical instruments. A good way to improve the reliability of clinical instruments is to repeat the same instruments many times [29], or collect a range of instruments measuring the same construct and use a combined score [124], or gain scores from multiple raters [23]. As discussed in section Recommendation 5: Combining multimodal neuroimaging and other data types, digital phenotyping opportunities such as mobile health applications offer opportunities for repeated measurements that also represent ecological momentary assessment to help avoid recall bias. Similar to the suggestions to improve the reliability of clinical instruments, repeated measures is the primary recommendation to improve the reliability of imaging measures. In particular, sufficiently long combined scan times for functional MRI (25+ minutes) potentially acquired across multiple sessions to control head motion enable more stable estimates of imaging measures [125, 126]. In addition, minimizing scanner and site effects promotes reliability. Lastly, more work is needed to assess the comparative reliability of imaging analysis pipelines and resulting summary measures to enable researchers and clinicians to make informed analytical choices that maximize reliability.

### Opportunity 3: Adjusting the academic culture to the era of big data

As a result of the increasing role and impact of big data, we are seeing a greater emphasis on team science due to the need for consortia, collaboration, and multidisciplinary skills. Simultaneously, we are also seeing the increased importance of open science practices such as data sharing, code sharing, preregistration, and the development of related tools, ontologies, and infrastructures. As the role of team science and open science grows, there is an important need for academic incentive structures to reflect the evolving academic landscape. In Box 3, we offer a set of suggestions targeted at academic power structures to achieve such aligned incentive structures. Encouragingly, many of these suggestions are already being adopted including requirements for data sharing from publishers [127] and funders [128] and broader publishing reform [129–131].

Box 3 Recommendations for academic incentive structures to value big data, team science, and open science **Journals and Publishers**

- Strongly encourage or require data and/or code sharing
- Encourage preregistration
- Offer registered report formats or sign on to the Peer Community In (PCI) Registered Reports (https://rr.peercommunityin.org/)
- Reward replicability and rigor over effect size and novelty
- Publish null findings and replication studies

**Funders**

- Support research across all study types shown in Table 1
- Create dedicated funding calls for big data/ secondary data analysis
- Create dedicated study sections/ review panels for big data/ secondary data analysis
- Consider open science and team science activities when evaluating the applicant
- Require data and/or code sharing
- Encourage budgeting of funds for personnel time and other resources needed for data sharing, harmonization, annotation, mapping, etc.

**Academic Institutions**

- Develop Standard Operating Procedures (SOPs) and provide sufficient resources for streamlined and timely DUA and MTA contract processing
- Support up-to-date computational resources and IT support (which requires competitive salaries to prevent “brain drain” into industry)
- Update tenure and promotion guidelines to explicitly capture open science and team science efforts

**Hiring Committees & Tenure and Promotions Committees**

- Consider open science activities such as data sharing, tool development, outreach, etc. when evaluating a candidate
- Value collaboration and team science (e.g., resulting in middle authorship papers and/or consortia grants) when evaluating a candidate
- Avoid simplistic metrics that may suffer from publication/citation bias (e.g., H-index, journal impact factor, publication count) in favor of a holistic review of a candidate
- Reward practices indicative of scientific rigor (e.g., replication) and open science (e.g., sharing)

**Grant and Paper Reviewers**

- Be realistic regarding the extensive personnel and resources required to acquire big data and perform secondary analysis studies using big data
- Avoid the assumption that all secondary analysis studies are “exploratory”
- Reward innovative use of big data

### Opportunity 4: Addressing remaining gaps in our approach to big data

The goal of this paper was to discuss current “best practice” approaches for using and collecting big datasets. However, large scale data in neuroimaging are still a relatively new frontier and there are many aspects of big data research where best practices are yet to develop. One open question is how to effectively budget for the personnel, storage, and compute infrastructure to ensure long-term sustainability of a big dataset, and how to secure long-term budgeting in the cyclic and grant-oriented academic landscape. Another open question is how to address the potential downsides of repeated re-use of big datasets in relation to cross-laboratory multiple comparisons, p-hacking, and hampered generalizability [48]. Out-of-sample prediction efforts that leverage multiple different big datasets in one research project may help to address some re-use concerns such as generalizability.

## Supplementary information

Supplemental Material
