# Supplementary material for: Current best practices and future opportunities for reproducible findings using large-scale neuroimaging in psychiatry
Source: Neuropsychopharmacology. 2024 Aug 8;50(1):37–51. doi: 10.1038/s41386-024-01938-8 (PMC11526024; doi:10.1038/s41386-024-01938-8)
Supplement: Supplementary file 1 — Supplemental Material [file 41386_2024_1938_MOESM1_ESM.docx]

Supplemental Material


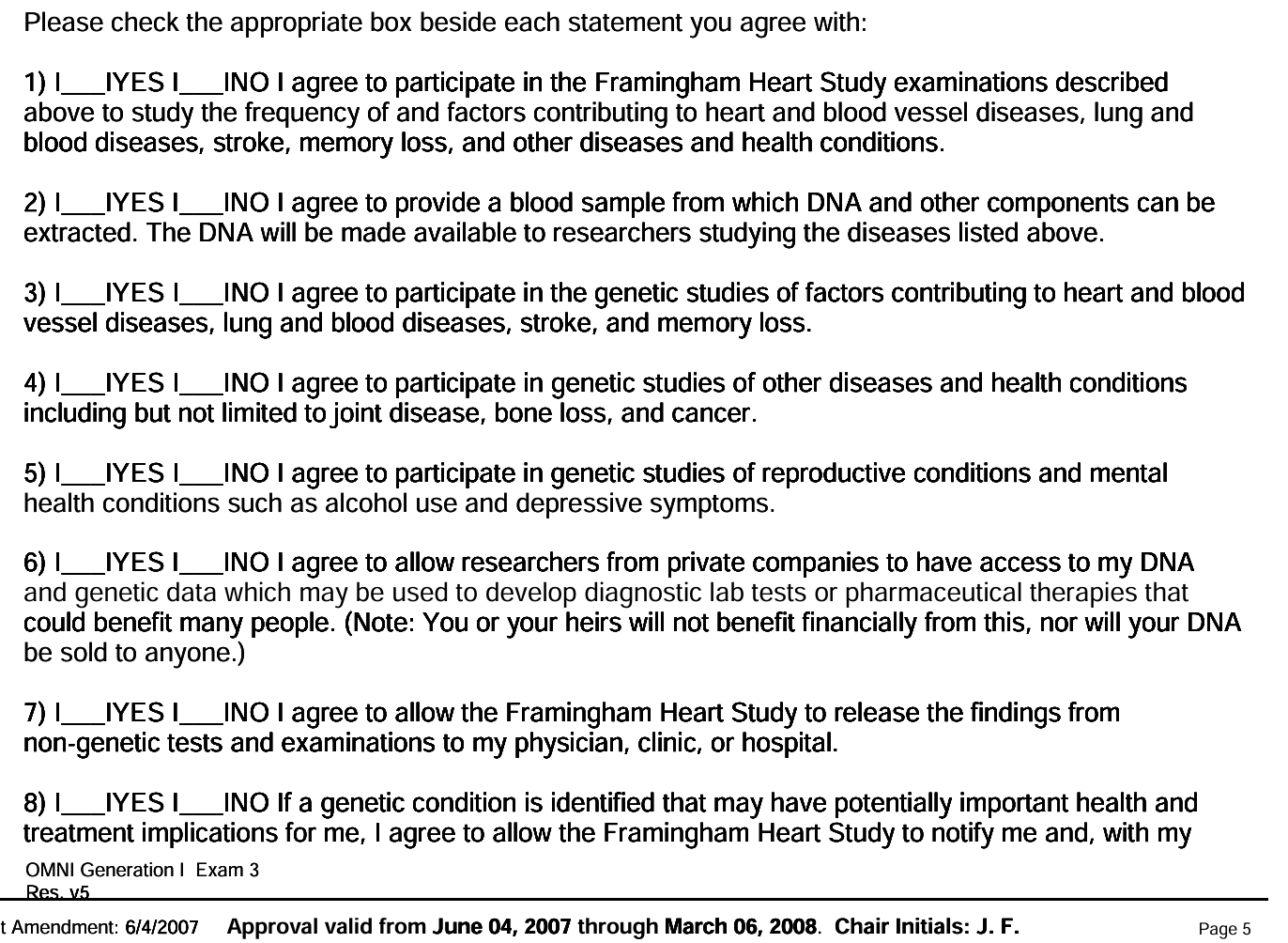


**Supplementary Figure 1.** A portion of the data use agreement from the Framingham Heart Study, which started in 1948, specifically asking participants to consent to different types of research. As some participants may have specific concerns with mental health research, due to stigma or other reasons, it can help to clearly document for what types of research the participants data may be used. From: [**https://www.framinghamheartstudy.org/files/2017/08/Omni-Gen-1-Cohort-Exam-3.pdf**](https://www.framinghamheartstudy.org/files/2017/08/Omni-Gen-1-Cohort-Exam-3.pdf)

| **Supplementary Box 1: Instrument Dictionary Example** | | | | | |
| --- | --- | --- | --- | --- | --- |
| **Domain** | **Instrument Long Name** | **Instrument Short Name** | **Acquisition Platform** | **version or modification** | **DOI** |
| Diet and Lifestyle | Pittsburgh Sleep Quality Index | psqi | REDCap |  | https://doi.org/10.1016/0165-1781(89)90047-4 |
| Diet and Lifestyle | Automated Self-Administered 24-Hour Dietary Assessment Tool | asa24 | ASA24 (https://epi.grants.cancer.gov/asa24/) |  | https://doi.org/10.1016/j.jfca.2009.02.003 |
| Cognition | Flanker Inhibitory Control and Attention Test | flanker | NIH Toolbox iPad Application (https://nihtoolbox.org/get-the-toolbox/) | v2.1 | https://doi.org/10.3758/BF03203267 |
| Emotion | Center for Epidemiologic Studies Depression Scale | cesd | REDCap |  | https://doi.org/10.1007/978-1-4419-1005-9_732 |
| Demographics | Family Demographics | demog | REDCap | unpublished; Study-specific | https://doi.org/10.1016/j.neuroimage.2018.10.009 |

| **Supplementary Box 2: Variable Dictionary Example** | | | | | | |
| --- | --- | --- | --- | --- | --- | --- |
| **Instrument Short Name** | **Variable** | **Label** | **Values or Calculations** | **Units** | **Missing Value Codes** | **Crosswalk to NDA**  **Structure:**  **Element** |
| psqi | psqi1 | 1. During the past month, when have you usually gone to bed at night? |  | military time (HH:MM) |  | psqi01:  psqip1 |
| psqi | psqi2 | 2. During the past month, how long has it usually taken you to fall asleep each night? |  | minutes |  | psqi01:  psqip2 |
| psqi | psqi3 | 3. During the past month, when have you usually gotten up in the morning? |  | military time (HH:MM) |  | psqi01:  psqip3 |
| psqi | psqi4 | 4. During the past month, how many hours of actual sleep did you get at night? (This may be different than the number of hours you spend in bed.) |  | hours |  | psqi01:  psqip4 |
| psqi | psqi5a | (a) Cannot get to sleep within 30 minutes | 0, Not during the past month \| 1, Less than once a week \| 2, Once or twice a week \| 3, Three or more times a week |  |  | psqi01:  psqip6a_1 |
| psqi | psqi5b | (b) Wake up in the middle of the night or early morning | 0, Not during the past month \| 1, Less than once a week \| 2, Once or twice a week \| 3, Three or more times a week |  |  | psqi01:  psqip6a_2 |
| psqi | psqi_global | Global PSQI Score:  Add the seven component scores together: | if([psqi_com1] <> "NaN" and [psqi_com2] <> "NaN" and [psqi_com3] <> "NaN" and [psqi_com4] <> "NaN" and [psqi_com5] <> "NaN" and [psqi_com6] <> "NaN" and [psqi_com7] <> "NaN", sum([psqi_com1], [psqi_com2], [psqi_com3], [psqi_com4], [psqi_com5], [psqi_com6], [psqi_com7]),"NaN") |  |  | psqi01:  psqi_total |
| flanker | Age_Corrected_Standard_Score | Age Corrected Standard score for Flanker Instrument (Inst) specified | https://www.nihtoolbox.org/app/uploads/2022/05/Toolbox_Scoring_and_Interpretation_Guide_for_iPad_v1.7-5.25.21.pdf |  |  | flanker01:  nih_flanker_ageadjusted |
| flanker | National_Percentile_Age_Adjusted | Age Adjusted National Percentile for Flanker Instrument (Inst) specified | https://www.nihtoolbox.org/app/uploads/2022/05/Toolbox_Scoring_and_Interpretation_Guide_for_iPad_v1.7-5.25.21.pdf |  |  | flanker01:  nih_flanker_natperc |
| flanker | RawScore | Raw Score for the Flanker Instrument (Inst) specified | https://www.nihtoolbox.org/app/uploads/2022/05/Toolbox_Scoring_and_Interpretation_Guide_for_iPad_v1.7-5.25.21.pdf |  |  | flanker01:  nih_flanker_raw |
| flanker | Uncorrected_Standard_Score | Uncorrected Standard score for Flanker Instrument (Inst) specified | https://www.nihtoolbox.org/app/uploads/2022/05/Toolbox_Scoring_and_Interpretation_Guide_for_iPad_v1.7-5.25.21.pdf |  |  | flanker01:  nih_flanker_unadjusted |
| flanker | Inst | NIH Toolbox Instrument Name and Version Number, per iPAD export |  |  |  | flanker01:  version_form |
| flanker | ItmCnt | Count of items to which participants responded |  |  |  | flanker01:  wcst_ni |
| demog | croms_income | Household Income Level (per year) |  | dollars per year | -9999, Missing \| -8888, Refused \| -7777, Unknown |  |

**Supplementary Table 1. Table of publicly available “Big Data” datasets available to the scientific research community in June, 2024.**

| **Dataset** | **Full name** | **Imaging Sample Size*** | **Study category** | **Additional Information** | **Reference / Website** |
| --- | --- | --- | --- | --- | --- |
| ABCD | Adolescent Brain and Cognitive Development | 11,000 | Epidemiological data | Longitudinal study of brain development and child health in the US | <https://abcdstudy.org/> |
| ABIDE | Autism Brain Imaging Data Exchange | 1100 | Retrospective big data | An aggregated collection of brain imaging data from laboratories around the world to study autism | <https://fcon_1000.projects.nitrc.org/indi/abide/> |
| ADNI | Alzheimer's Disease Neuroimaging Initiative | 2000+ | Prospective big data | Longitudinal multi-phase study of brain aging, neurodegeneration, and Alzheimer's disease | <https://adni.loni.usc.edu/> |
| AIBL | Australian Imaging, Biomarker and Lifestyle | 3000 | Prospective big data | Longitudinal study of aging and Alzheimer's disease | <https://aibl.org.au/> |
| ALSPAC | Avon Longitudinal Study of Parents and Children | 14,000 | Epidemiological data | Longitudinal multigeneration birth cohort study that recruited pregnant women in the early 1990s | <https://www.bristol.ac.uk/alspac/> |
| CNEuroMod | Courtois Project on Neuronal Modelling | 6 | Precision data | 500 hours of functional data per subject | <https://www.cneuromod.ca/> |
| CoRR | Consortium for Reliability and Reproducibility | 1600 | Precision data / Retrospective big data | Numerous test-retest and traveling subjects databases | <https://fcon_1000.projects.nitrc.org/indi/CoRR/html/> |
| GUSTO | Growing Up in Singapore Towards healthy Outcomes | 1,400 | Prospective big data | Longitudinal study of mother-children diads across Singapore | <https://gustodatavault.sg/home> |
| HBCD | Healthy Brain and Child Development | 7500 | Prospective big data | Long term study of early brain development in the US | <https://hbcdstudy.org/> |
| HBN | Healthy Brain Network | 4,000 | Prospective big data | Community-centered program in the greater New York area that collects data from children and adolescents at risk for mental illness | <https://fcon_1000.projects.nitrc.org/indi/cmi_healthy_brain_network/index.html> |
| HCP | Human Connectome Project | - |  | In addition to the larger young adult, lifespan, aging, and development HCP projects, the HCP network of datasets also includes many accessable datasets on a variety of specific studies, many of sample sizes between 200 and 800 individuals, including individuals with or at risk for psychiatric conditions, undergoing interventions, and other specific populations (for sample infants, Amish) | <https://www.humanconnectome.org/> |
| HCP-YA | Human Connectome Project Young Adult | 1200 | Prospective big data |  |  |
| HCP-Aging |  | 1200 | Prospective big data |  |  |
| HCP-Development |  | 1300 | Prospective big data |  |  |
| LD-HCP | Lifespan Developing Human Connectome Project | 1500 | Prospective big data |  |  |
| HABS-HD | Health and Aging Brain Study - Health Disparities | 3000 | Prospective big data | Longitudinal study of aging and Alzheimer's disease in racial and ethnically diverse and under-represented populations | <https://apps.unthsc.edu/itr/research> |
| HUNT | Trøndelag Health Study | 1000 | Epidemiological data | Questionnaire data, clinical measurements and samples from over 250,000 Norweigen inhabitants since 1984 | <https://www.ntnu.edu/hunt/> |
| Midnight Scan Club |  | 10 | Precision data | Large amount of per-individual data with 12 two hour scan sessions per subject | <https://openneuro.org/datasets/ds000224/versions/1.0.4> |
| NKI-Rockland | Nathan Klien Institute Rockland | 1500 | Prospective big data | Research program aiming to study brain/behavior development and changes over the course of life (age 6 to 85 years) | <http://rocklandsample.org/> |
| OASIS | Open Access Series of Imaging Studies | 2000+ | Retrospective big data | Multiple phases of studies aimed at making neuroimaging data sets freely available | <https://sites.wustl.edu/oasisbrains/> |
| PING | Pediatric Imaging, Neurocognition, and Genetics | 1400 | Prospective big data | Multi-site study of pediatric and adolescent development | <https://chd.ucsd.edu/research/ping-study.html> |
| PNC | Philadelphia Neurodevelopmental Cohort | 1400 | Prospective big data | Population based study of neurodevelopment in individuals aged 8-21 to study brain, behavior, and the role of genetics | <https://www.med.upenn.edu/bbl/philadelphianeurodevelopmentalcohort.html> |
| PPMI | Parkinson's Progression Markers Initiative | 4000 | Prospective big data | Multi-site in depth study of Parkinson disease progression, including patients, unaffected controls, and prodromal individuals | <https://www.ppmi-info.org/> |
| QTIM | Queensland Twin IMaging | 1000 | Prospective big data | Study of monozygotic and dizygotic young adult twins | [https://openneuro.org/datasets/ds004169/](https://openneuro.org/datasets/ds004169/versions/1.0.6) |
| UK Biobank | UK Biobank | 100,000 | Epidemiological data | Large-scale epidemiological study of over 500,000 UK residents | <https://www.ukbiobank.ac.uk/> |
|  |  |  |  |  |  |
| * | Study sample sizes may differ from those imaged, as imaging may only be conducted on a subset of participants for some studies. If a study is ongoing, the target sample size is listed. | | | | |
